# Health-related quality of life in children with HIV/AIDS: Child and parent perspectives at a tertiary ART clinic in Kathmandu, Nepal

**DOI:** 10.1371/journal.pone.0346012

**Published:** 2026-03-30

**Authors:** Kamala Budha, Prakash Ghimire

**Affiliations:** 1 Paediatric Unit, Seti Provincial Hospital, Kailali, Nepal; 2 Public Health Section, Chandragiri Municipality Office, Kathmandu, Nepal; Bogomolets National Medical University: Nacional'nij medicnij universitet imeni O O Bohomol'ca, UKRAINE

## Abstract

**Background:**

Children living with human immunodeficiency virus/acquired immunodeficiency syndrome (HIV/AIDS) face persistent physical, emotional, and social challenges that can reduce their quality of life. In Nepal, evidence describing the health-related quality of life (HRQoL) of this population remains limited.

**Objective:**

To assess health-related quality of life and the level of agreement between child self-reports and parent proxy reports among children living with HIV/AIDS receiving antiretroviral therapy at a tertiary care hospital in Kathmandu, Nepal.

**Methods:**

An analytical cross-sectional study was conducted from July to September, 2022 among 105 children aged 6–18 years and their parents attending the antiretroviral therapy clinic at Tribhuvan University Teaching Hospital. HRQoL was assessed using the Pediatric Quality of Life Inventory Version 4.0 Generic Core Scales which comprises physical, emotional, social, and school functioning domains, for both child self-reports and parent proxy reports. Descriptive statistics summarized participant characteristics and HRQoL scores. Chi-square and Fisher’s exact tests examined associations between sociodemographic variables and quality of life. Intraclass correlation coefficients (ICC) were calculated to assess agreement between child self-reports and parent proxy reports.

**Results:**

More than half of the children (58.1%) had poor HRQoL. The mean total HRQoL score from child self-reports was 68.8 ± 5.7, with higher scores in the social (73.5 ± 8.1) and physical (73.3 ± 6.1) domains compared to the emotional (65.1 ± 8.9) and school functioning (62.8 ± 7.5) domains. Parent proxy reports showed slightly higher mean scores (70.4 ± 3.7). Overall agreement between child self-reports and parent proxy reports was poor (ICC = 0.405), with the highest agreement observed in the physical functioning domain.

**Conclusion:**

Poor quality of life is common among children living with HIV/AIDS in Nepal, particularly in emotional and school functioning domains. Targeted interventions addressing psychosocial well-being and educational participation are needed to improve overall quality of life in this group.

## Introduction

Human immunodeficiency virus/acquired immunodeficiency syndrome (HIV/AIDS) remains a major global health concern, often accompanied by stigma and discrimination that negatively affect quality of life [1,2]. The World Health Organization (WHO) South-East Asia Region (SEAR) bears the second highest burden of HIV after sub-Saharan Africa, with approximately 120,000 children and adolescents aged 0–19 years living with HIV/AIDS [3,4]. Nepal, a developing country sharing an open border with India, which has the highest HIV prevalence in SEAR, faces a similar public health challenge [4].

HIV infection in children is a chronic condition requiring lifelong antiretroviral therapy [5]. Children living with HIV are at increased risk of low self-esteem, behavioral and emotional problems, and impaired social functioning, all of which can reduce quality of life [6]. As a result, assessment of health-related quality of life (HRQoL) has become an important outcome alongside traditional biomedical indicators, particularly as improved access to ART has increased survival into adolescence and adulthood [5].

Evidence from India, China, and other countries in South and Southeast Asia indicates that children with HIV generally report lower quality of life compared with uninfected peers, particularly in emotional and school functioning domains [2,7–9]. Research has shown that stigma, psychosocial stress, treatment burden, and limited social support play important roles in shaping reduced quality of life among this population [6,7,9]. Similar findings have also been reported in studies involving children with other chronic illnesses, where long-term disease management negatively affects multiple dimensions of quality of life [10].

Assessment of HRQoL in pediatric populations commonly incorporates both child self-reports and parent proxy reports [6,11]. However, previous studies have demonstrated variable levels of agreement between children and their parents regarding perceived quality of life. Agreement is often higher for observable domains such as physical functioning, while lower agreement has been reported for less observable domains such as emotional, social, and school functioning [6,8]. These discrepancies suggest that parents may not always fully perceive children’s subjective experiences, highlighting the importance of including both child and parent perspectives in HRQoL assessment [6,11].

Current management strategies for children living with HIV emphasize not only clinical treatment but also the improvement of quality of life [8,9]. Timely assessment of HRQoL provides valuable insights into overall health status and may guide interventions to maintain or enhance well-being [12]. However, in Nepal, research on the quality of life of children living with HIV/AIDS remains limited, and evidence on the level of agreement between child self-reports and parent proxy reports is scarce. Therefore, this study aimed to assess the quality of life of children receiving ART at a tertiary hospital in Nepal and to examine the association of sociodemographic and clinical factors with quality of life, as well as the agreement between child self-reports and parent proxy reports.

## Materials and methods

An institutional-based cross-sectional study was conducted from 01 July 2022 to30 September 2022 to assess the quality of life of children living with HIV/AIDS receiving antiretroviral therapy (ART) at the ART center of Tribhuvan University Teaching Hospital (TUTH), Maharajgunj, Kathmandu, Nepal. TUTH is a 700-bed national referral public hospital established in 1982 and has been providing ART services for children since 2005. The study aimed to evaluate the quality of life of children living with HIV/AIDS, examine associations between sociodemographic and clinical factors and quality of life, and assess agreement between child self-reports and parent proxy reports.

The study population included children aged 6–18 years registered at the ART center and receiving ART, along with their parents. Children and parents who were seriously ill or unable to respond were excluded. A total of 105 children who attended the ART center during the study period were included. Since the overall number of eligible children was limited, a census approach was used, ensuring complete coverage of the population and eliminating sampling error. Written informed consent was obtained from parents, along with assent from children under 18 years of age. Participants’ privacy and dignity were ensured, and they were allowed to withdraw from the study at any time.

Data were collected using a structured interview schedule developed based on the study objectives, literature review, previous studies, and consultation with subject experts. The questionnaire included sociodemographic characteristics (age, sex, educational status, and area of residence), clinical information (duration of ART and WHO clinical stage), and quality of life assessment using the Pediatric Quality of Life Inventory (PedsQL) 4.0 Generic Core Scales [11]. The PedsQL consists of 23 items across four domains: physical, emotional, social, and school functioning. Age-specific validated versions of the PedsQL were used for children aged 6–12 years and 13–18 years, and the instrument was administered to both children (self-report) and one parent or primary caregiver (proxy report) accompanying the child during the clinic visit [11].

Items were scored on a 5-point Likert scale (0 = never a problem; 4 = almost always a problem) and transformed to a 0–100 scale, with higher scores indicating better quality of life. For descriptive interpretation, impaired HRQoL was defined as a total PedsQL score of <70. This threshold was informed by anchor-based cutoff scores established by Huang et al. [13], who identified a total score of approximately 70 among children aged ≥8 years as optimally discriminating those with major chronic health conditions from healthier peers. Given that HIV is a chronic condition and that all participants in this study were aged ≥8 years, this threshold was applied for descriptive categorization.

A Nepali-language version of the Pediatric Quality of Life Inventory™ (PedsQL™), obtained through authorized translation sources and previously used in pediatric studies in Nepal, was utilized in this study [14,15]. This instrument has demonstrated strong reliability, with Cronbach’s alpha value exceeding 0.70 for all subscales in multiple prior studies [11,16]. Face-to-face interviews were conducted by the researcher, with four to six participants interviewed per day; each interview lasted 20–30 minutes. Privacy was maintained by conducting interviews individually, and all data were reviewed for completeness immediately after collection. Sociodemographic characteristics of parents were not collected, which is acknowledged as a limitation of the study.

Data were entered into SPSS version 16.0 and analyzed using descriptive statistics, including frequency, percentage, mean, and standard deviation. Associations between independent variables and quality of life were examined using Chi-square or Fisher’s exact tests, as appropriate. Agreement between child self-reports and parent proxy reports was assessed by intraclass correlation coefficients (ICC). ICC values were interpreted as poor (<0.50), moderate (0.50–0.75), and good (0.75–0.90), and excellent (>0.90), based on established guidelines [17].

Ethical approval for this study was obtained from the Institutional Review Committee of Tribhuvan University Teaching Hospital (TUTH) (Approval No: 490(6–11)E2), and written permission was obtained from the Department of Internal Medicine, TUTH. Written informed consent was obtained from the parents or legal guardians of all participating children, and assent was obtained from children who were capable of providing it, prior to enrollment. Only non-invasive procedures were used, confidentiality was strictly maintained, and all data were stored securely on a password-protected device accessible only to the research team.

## Results

A total of 105 children living with HIV/AIDS receiving ART and their parents participated in the study. The mean age of the children was 13.79 ± 3.13 years, ranging from 8 to 18 years, with 60% aged 13–18 years and 40% aged 8–12 years. Slightly more than half of the respondents were female (51.4%), and all participants resided within the Kathmandu Valley. Nearly three-fourths (75.2%) had attained secondary-level education, while only 10.5% had completed SLC or higher (Table 1).

**Table 1:** Socio-demographic characteristics of respondents (n = 105).

Characteristics	Frequency(N)	Percentage (%)
Age (in completed years)		
8-12	42	40.0
13-18	63	60.0
Gender		
Male	51	48.6
Female	54	51.4
Educational Level		
Primary	15	14.3
Secondary	79	75.2
Higher Secondary and Above	11	10.5

All children had been receiving ART for more than two years. Most participants (71.4%) were classified in the second WHO clinical stage, and 28.6% were in the third stage of HIV infection (Table 2).

**Table 2:** Clinical characteristics of the respondents (n = 105).

Characteristics	Frequency (N)	Percentage (%)
Duration of ART		
≤2years	0	0.0
>2years	105	100.0
WHO clinical staging		
1^st^ stage	0	0.0
2^nd^ stage	75	71.4
3^rd^ stage	30	28.6
4^th^ stage	0	0.0

**Fig 1:**
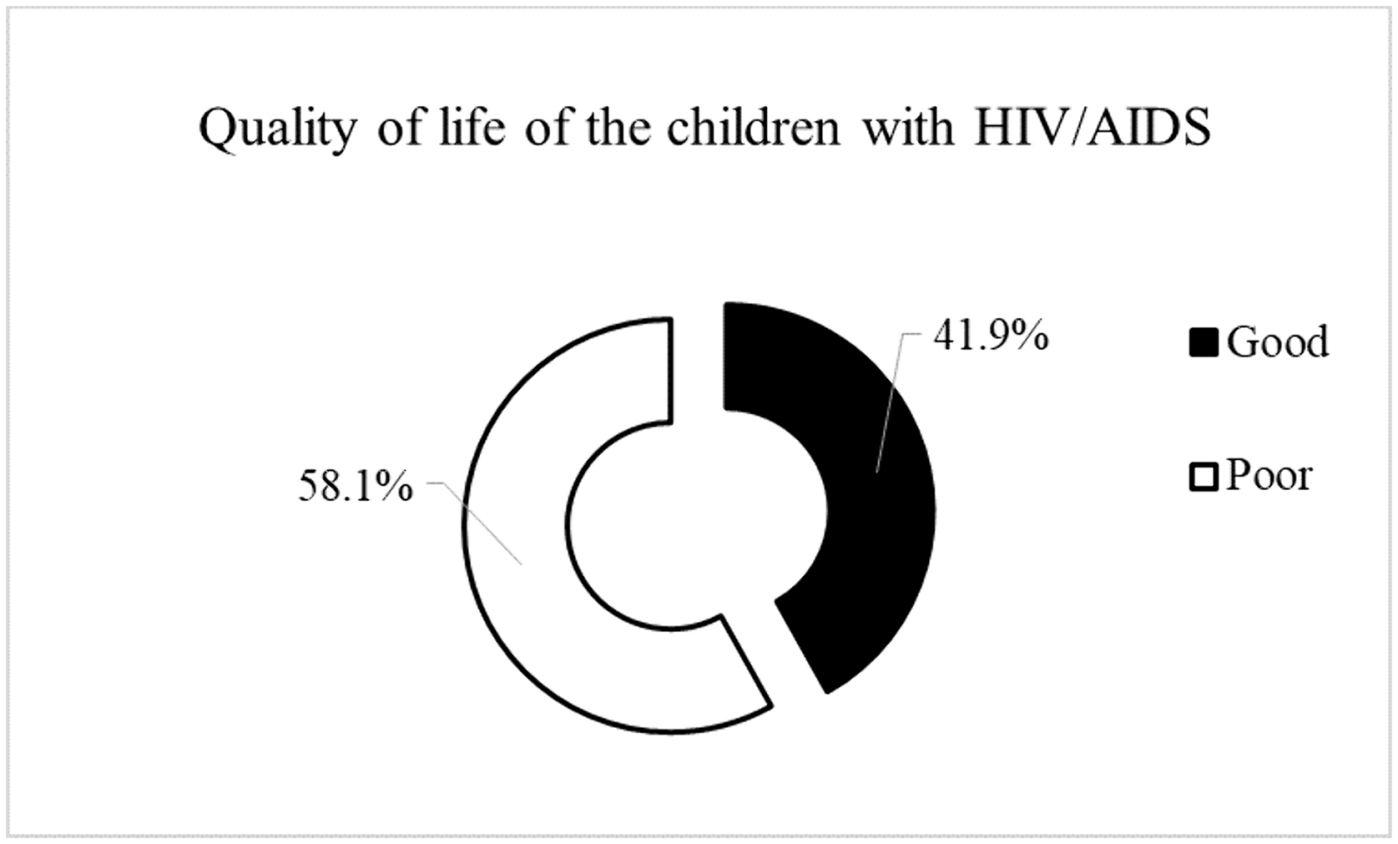
Quality of life of the respondents.

Regarding quality of life, more than half of the children (58.1%) were categorized as having poor quality of life, whereas 41.9% had good quality of life (Fig 1).

The mean total score of the Pediatric Quality of Life Inventory (PedsQL) 4.0 was slightly higher in parent proxy reports (70.4 ± 3.7) than child self-reports (68.8 ± 5.7). For child self-reports, the highest mean score was observed in the social functioning domain (73.5 ± 8.1), followed by physical functioning (73.3 ± 6.1), whereas the lowest score was in the school functioning domain. Similarly, parents reported the highest scores in physical functioning (74.4 ± 4.9) followed by social functioning (74.3 ± 2.7), with the lowest scores in school functioning (Table 3).

**Table 3:** Quality of life score from child self-report and parents proxy report (n = 105).

PedsQL Scale	Child self-report		Parents proxy report	
Domain	Mean	SD	Mean	SD
Total score	68.8	5.7	70.4	3.7
Physical functioning	73.3	6.1	74.4	4.9
Emotional functioning	65.1	8.9	68.3	7.7
Social functioning	73.5	8.1	74.3	2.7
School functioning	62.8	7.5	64.8	4.8

Age-stratified analysis of child self-reports revealed that children aged 8–12 years had higher total mean scores (69.8 ± 2.7) compared to those aged 13–18 years (68.3 ± 4.1). Domain-specific scores were generally higher in the younger age group except for school functioning. In both age groups, the social domain had the highest scores, followed by physical functioning (Table 4).

**Table 4:** Age and domain wise mean score of child self-report on quality of life (n = 105).

Age category	Physical domain	Emotional domain	Social domain	School functioning	Total Quality of life
8-12 years					
Mean	73.8	67.7	74.3	64.6	69.8
SD	4.9	6.6	5.8	7.1	2.7
N	42	42	42	42	42
13-18 years					
Mean	72.9	64.1	73.0	61.5	68.3
SD	6.8	10.1	9.3	7.5	4.1
N	63	63	63	63	63

Table 5 shows that the overall mean scores of parents’ proxy-reports on children’s quality of life were almost similar in both age groups 8–12 years (70.5 ± 3.1) and 13–18 years (70.4 ± 4.1). Across domains, parents of younger children (8–12 years) reported slightly higher quality of life than older children, except in the physical functioning domain. The highest mean scores in both age groups were in the physical domain (74.0 ± 4.3 and 74.7 ± 5.4, respectively), followed by the social domain (74.5 ± 1.8 and 74.1 ± 3.1, respectively).

**Table 5:** Age and domain wise mean score of parent's proxy report of Quality of life (n = 105).

Age category	Physical domain	Emotional domain	Social domain	School functioning	Total Quality of life
8-12 years					
Mean	74.0	68.5	74.5	64.9	70.5
SD	4.3	7.1	1.8	3.4	3.1
N	42	42	42	42	42
13-18 years					
Mean	74.7	68.2	74.1	64.8	70.4
SD	5.4	7.7	3.1	5.5	4.1
N	63	63	63	63	63

Children’s responses on the Pediatric Quality of Life Inventory (PedsQL 4.0) are presented in Table 6. In the physical functioning domain, the most frequently reported issue was low energy, with 42.9% of children responding “sometimes.” In the emotional functioning domain, 56.2% of children reported “sometimes” feeling worried about what would happen to them, and the same proportion reported feeling angry; 41.9% reported sometimes feeling sad.

**Table 6:** Frequency of children’s self-responses on Pediatric Quality of Life Inventory (PedsQL 4.0) (n = 105).

Domain	Never N (%)	Almost never N (%)	Sometimes N (%)	Often N (%)	Almost always N (%)
Physical functioning					
It is hard for me to walk more than one block	10 (9.5)	93 (88.6)	2 (1.9)	0 (0.0)	0 (0.0)
It is hard for me to run	9 (8.6)	87 (82.9)	9 (8.6)	0 (0.0)	0 (0.0)
It is hard for me to do sports activity or exercise	6 (5.7)	85 (81.0)	14 (13.3)	0 (0.0)	0 (0.0)
It is hard for me to lift something heavy	3 (2.8)	86 (81.9)	14 (13.3)	1 (1.0)	1 (1.0)
It is hard for me to take a bath or shower by myself	8 (7.6)	96 (91.4)	1 (1.0)	0 (0.0)	0 (0.0)
It is hard for me to do chores around the house	9 (8.6)	95 (90.4)	1 (1.0)	0 (0.0)	0 (0.0)
I hurt or ache	3 (2.8)	85 (81.0)	17 (16.2)	0 (0.0)	0 (0.0)
I have low energy	2 (1.9)	58 (55.2)	45 (42.9)	0 (0.0)	0 (0.0)
Emotional functioning					
I feel afraid or scared	4 (3.8)	73 (69.5)	28 (26.7)	0 (0.0)	0 (0.0)
I feel sad or blue	3 (2.8)	57 (54.3)	44 (41.9)	1 (1.0)	0 (0.0)
I feel angry	1 (1.0)	44 (41.9)	59 (56.1)	1 (1.0)	0 (0.0)
I have trouble sleeping	6 (5.7)	81 (77.1)	17 (16.2)	1 (1.0)	0 (0.0)
I worry about what will happen to me	3 (2.8)	42 (40.0)	59 (56.2)	1 (1.0)	0 (0.0)
Social functioning					
I have trouble getting along with other teens	6 (5.7)	93 (88.6)	5 (4.7)	1 (1.0)	0 (0.0)
Other teens do not want to be my friend	4 (3.8)	94 (89.5)	5 (4.8)	2 (1.9)	0 (0.0)
Other teens tease me	6 (5.7)	92 (87.6)	7 (6.7)	0 (0.0)	0 (0.0)
I cannot do things that other teens my age can do	8 (7.6)	84 (80.0)	12 (11.4)	1 (1.0)	0 (0.0)
It is hard to keep up with my peers	8 (7.6)	73 (69.5)	22 (21.0)	2 (1.9)	0 (0.0)
School functioning					
It is hard to pay attention in class	2 (1.9)	76 (72.4)	22 (21.0)	5 (4.7)	0 (0.0)
I forget things	0 (0.0)	29 (27.6)	73 (69.5)	3 (2.9)	0 (0.0)
I have trouble keeping up with my schoolwork	4 (3.8)	77 (73.3)	24 (22.9)	0 (0.0)	0 (0.0)
I miss school because of not feeling well	2 (1.9)	72 (68.6)	29 (27.6)	2 (1.9)	0 (0.0)
I miss school to go to the doctor or hospital	0 (0.0)	8 (7.6)	97 (92.4)	0 (0.0)	0 (0.0)

In the social functioning domain, 21.0% reported sometimes having difficulty keeping up with peers, and 11.4% reported being unable to do things that other children their age could do. In the school functioning domain, 27.6% of children reported sometimes missing school due to not feeling well, and 92.4% reported missing school to attend doctor or hospital visits. Difficulties in paying attention (21.0%) and keeping up with schoolwork (22.9%) were also reported.

As presented in Table 7, parents most frequently reported problems in the emotional domain, with over half (51.4%) indicating their child sometimes felt angry and 40.9% reporting worry about what would happen to them. In the physical domain, 16.2% reported low energy and 8.6% noted aches. Difficulties in social relationships were less common, with only 6.7% reporting problems keeping up with peers. In the school domain, attention difficulties (10.5%) and forgetfulness (84.7%) were reported, while almost all parents (97.1%) indicated their child sometimes missed school due to medical appointments.

**Table 7:** Frequency of Parent’s response on pediatric quality of life inventory tools version 4.0 (n = 105).

Domain	Never N (%)	Almost never N (%)	Sometimes N (%)	Often N (%)	Almost always N (%)
Physical functioning					
Walking more than one block	4 (3.8)	100 (95.2)	1 (1.0)	0 (0.0)	0 (0.0)
Running	4 (3.8)	97 (92.4)	4 (3.8)	0 (0.0)	0 (0.0)
Participating in sports activity or exercise	2 (1.9)	101 (96.2)	2 (1.9)	0 (0.0)	0 (0.0)
Lifting something heavy	3 (2.8)	97 (92.4)	5 (4.8)	0 (0.0)	0 (0.0)
Taking a bath or shower by him or herself	3 (2.8)	101 (96.2)	0 (0.0)	1 (1.0)	0 (0.0)
Doing chores around the house	3 (2.8)	101 (96.2)	0 (0.0)	1 (1.0)	0 (0.0)
Having hurts or aches	1 (1.0)	95 (90.4)	9 (8.6)	0 (0.0)	0 (0.0)
Low energy level	1 (1.0)	87 (82.9)	17 (16.2)	0 (0.0)	0 (0.0)
Emotional functioning					
Feeling afraid or scared	3 (2.8)	85 (81.0)	17 (16.2)	0 (0.0)	0 (0.0)
Feeling sad or blue	2 (1.9)	72 (68.6)	31 (29.5)	0 (0.0)	0 (0.0)
Feeling angry	1 (1.0)	50 (47.6)	54 (51.4)	0 (0.0)	0 (0.0)
Trouble sleeping	1 (1.0)	102 (97.1)	2 (1.9)	0 (0.0)	0 (0.0)
Worrying about what will happen to him or her	1 (1.0)	60 (57.1)	43 (40.9)	1 (1.0)	0 (0.0)
Social functioning					
Getting along with other teens	0 (0.0)	104 (99.0)	1 (1.0)	0 (0.0)	0 (0.0)
Other teens not wanting to be his or her friend	0 (0.0)	104 (99.0)	1 (1.0)	0 (0.0)	0 (0.0)
Getting teased by other teens	0 (0.0)	104 (99.0)	1 (1.0)	0 (0.0)	0 (0.0)
Not able to do things that other teens his or her age can do	0 (0.0)	100 (95.2)	5 (4.8)	0 (0.0)	0 (0.0)
Keeping up with other teens	0 (0.0)	98 (93.3)	7 (6.7)	0 (0.0)	0 (0.0)
School functioning					
Paying attention in class	2 (1.9)	92 (87.6)	11 (10.5)	0 (0.0)	0 (0.0)
Forgetting things	1 (1.0)	15 (14.3)	89 (84.7)	0 (0.0)	0 (0.0)
Keeping up with schoolwork	2 (1.9)	93 (88.6)	10 (9.5)	0 (0.0)	0 (0.0)
Missing school because of not feeling well	1 (1.0)	96 (91.4)	8 (7.6)	0 (0.0)	0 (0.0)
Missing school to go to the doctor or hospital	0 (0.0)	3 (2.9)	102 (97.1)	0 (0.0)	0 (0.0)

Table 8 shows that children’s quality of life was not significantly associated with age, gender, educational status, and WHO clinical stage.

**Table 8:** Association between quality of life and characteristics of the respondents (n = 105).

Characteristics	Quality of Life		Chi-square/fisher exact	p value
	Poor N (%)	Good N (%)		
Age (in completed years)				
8-12	22	20	0.939	0.333
13-18	39	24		
Gender				
Male	31	20	0.295	0.587
Female	30	24		
Child’s educational status				
Primary level	9	6	0.080^a^	0.961
Secondary level	46	33		
SLC or above	6	5		
WHO clinical staging				
2^nd^ stage	41	34	1.268	0.260
3^rd^ stage	20	10		

^a^fisher exact test.

Intraclass correlation analysis demonstrated a statistically significant but poor level of agreement between child self-reports and parent proxy reports for total quality of life (ICC = 0.405, 95% CI:0.136–0.592, p = 0.002). Moderate agreement was observed for physical functioning (ICC = 0.613, 95% CI:0.432–0.736, p < 0.001) and emotional functioning (ICC = 0.523, 95% CI: 0.300–0.675, p < 0.001). In contrast, no statistically significant agreement was found for social functioning (ICC = 0.188, 95% CI: −0.195–0.448, p = 0.145) and school functioning (ICC = 0.172, 95% CI: −0.195–0.448, p = 0.145) (Table 9).

**Table 9:** Intraclass Correlation between Child self-report and Parents proxy report (n = 105).

Domain	ICC	95% confidence interval		p value
		Lower	Upper	
Physical functioning	0.613	0.432	0.736	<0.001*
Emotional functioning	0.523	0.300	0.675	<0.001*
Social functioning	0.188	−0.195	0.448	0.145
School functioning	0.172	−0.195	0.448	0.145
Total score	0.405	0.136	0.592	0.002*

*significant at p value<0.05.

## Discussion

HIV/AIDS remains a major public health issue among children and adults globally. Children living with HIV are at risk for low self-esteem, as well as emotional, behavioral, and social functioning problems, all of which may negatively impact quality of life (QoL) [6,8]. Understanding the QoL of these children is essential for guiding interventions to improve their overall health and well-being. This quantitative cross-sectional study assessed the QoL of 105 children aged 6–18 years receiving ART at a national level ART center in Kathmandu, Nepal.

In this study, 41.9% of children were categorized as having good QoL, while 58.1% had poor QoL. Thus, although a considerable proportion of children reported good QoL, more than half continued to experience substantial challenges. These findings contrast with a study from South India, where only 40% of children reported good QoL, and 60% had poor QoL [2]. Several factors may contribute to these differences, including access to ART, social support, and the psychosocial environment. In Nepal, children living with or affected by HIV/AIDS may experience stigma, discrimination, and changes in caregiving arrangements, including care by extended family members following parental illness or death, factors that may adversely influence their quality of life [18].

The total mean score of the PedsQL was slightly higher in parent proxy reports (70.44 ± 3.69) than child self-reports (68.78 ± 5.70), consistent with the findings of Lang T et al., where parents rated their children’s QoL higher than the children themselves (83 ± 10vs.78 ± 14) [19]. However, this finding contrasts with that reported by Adnyana IS et al., in which children reported higher quality of life scores than their parents (79.27 ± 9.61 vs. 75.76 ± 16.83) [8]. These discrepancies may reflect cultural differences, parental expectations, coping mechanisms, or variations in illness perception and chronicity across settings.

Domain-specific analysis revealed that children rated social functioning highest (73.52 ± 8.05) followed by physical functioning (73.27 ± 5.70), while parents rated physical functioning highest (74.40 ± 4.99) followed by social functioning (74.28 ± 2.7). School functioning consistently scored lowest in both reports. These patterns suggest that while physical health and social interaction may be relatively preserved, emotional well-being and school participation remain key areas of concern. Similar trends were observed in a South Indian study by Lang T et al., although their absolute scores were higher [19].

Age-stratified analysis showed that younger children (8–12 years) had slightly higher QoL scores than adolescents (13–18 years) across most domains, except school functioning. This is consistent with previous studies in Nigeria by Salako et al., which also reported higher social domain scores in younger children [6]. The lower QoL scores in older children may reflect increased awareness of HIV status, fear of stigma, and concerns regarding their long-term health and social relationships.

No significant associations were observed between QoL and sociodemographic factors (age, gender, education) or clinical stage in this study. Previous studies have reported demographic differences in quality of life among children living with HIV, including higher QoL scores among male children [6] and age-related variations across childhood and adolescence [19], although findings have been inconsistent across settings. Such differences may be attributed to variation in sample size, study design, or the availability of supportive services within ART programs.

Regarding parent–child agreement, overall concordance between child self-reports and parent proxy reports was poor (ICC = 0.405). Domain-specific analysis demonstrated moderate agreement for physical and emotional functioning, whereas agreement for social and school functioning was low and not statistically significant. This pattern suggests that parents may have greater difficulty perceiving children’s experiences in less observable psychosocial domains. Similar discrepancies have been reported in studies of children with other chronic illnesses, indicating that parent–child disagreement in HRQoL assessment is not unique to HIV and likely reflects inherent challenges in capturing subjective aspects of well-being through proxy reporting [10]. Similar patterns have also been reported in earlier PedsQL-based study in India among children living with HIV [19].

Overall, this study underscores the multidimensional nature of QoL in children living with HIV/AIDS and highlights the importance of including both child and parent perspectives in clinical assessment. Routine assessment of HRQoL alongside clinical monitoring may help healthcare providers identify psychosocial and educational challenges that are not apparent through biomedical indicators alone. Integrating HRQoL screening into pediatric ART services could facilitate timely referral for counselling, school-based support, and family-centered interventions.

Strengths of this study include the use of an internationally validated PedsQL tool and the inclusion of both child and parent reports. Limitations include its cross-sectional design, which precludes causal inference, and its single-center setting, which may limit generalizability. Self-reported measures may also be influenced by social desirability bias. Additionally, sociodemographic characteristics of parents were not collected, which may have influenced proxy reporting. Although gender differences in HRQoL have been reported in previous studies, the present study was not powered to conduct gender-stratified analyses. Similarly, agreement between child and parent reports was assessed for the overall sample, and subgroup analyses by age or gender were not performed due to limited sample sizes.

Future research should explore longitudinal changes in QoL, psychosocial determinants of well-being, and interventions targeting school participation, emotional support, and social functioning among children living with HIV in Nepal. Future studies with larger samples should also examine age- and gender-specific differences in HRQoL and child–parent agreement to further inform patient-centered HIV care.

## Conclusion

This study highlights that a considerable proportion of children living with HIV/AIDS experience poor health-related quality of life, with emotional and school functioning emerging as the most affected domains despite relatively preserved physical and social functioning. The poor concordance between child self-reports and parent proxy reports underscores the necessity of incorporating both perspectives to more accurately capture children’s lived experiences. These findings emphasize the importance of integrating routine psychosocial assessment, educational support, and family-centered interventions into pediatric HIV care to improve overall quality of life.
